# *TBK1* haploinsufficiency results in changes in the K63-ubiquitination profiles in brain and fibroblasts from affected and presymptomatic mutation carriers

**DOI:** 10.1007/s00415-021-10887-x

**Published:** 2021-11-20

**Authors:** Behzad Khoshnood, Abbe Ullgren, Jose Laffita-Mesa, Linn Öijerstedt, Kalicharan Patra, Inger Nennesmo, Caroline Graff

**Affiliations:** 1 Division for Neurogeriatrics, Centre for Alzheimer Research, Department of Neurobiology, Care Sciences and Society, Karolinska Institutet, Stockholm, Sweden; 2 Unit for Hereditary Dementias, Karolinska University Hospital Solna, Stockholm, Sweden; 3 Swedish FTD Initiative, Stockholm, Sweden; 4 Department of Pathology, Karolinska University Hospital, Stockholm, Sweden

**Keywords:** Frontotemporal dementia, Neurodegeneration, Ubiquitination, TBK1, Haploinsufficiency, Autophagy, FTD, FTLD, ALS

## Abstract

**Background:**

Frontotemporal dementia (FTD) is a neurodegenerative disease, resulting in progressive problems in language and/or behaviour and is often diagnosed before 65 years of age. Ubiquitin positive protein aggregates in the brain are among the key pathologic hallmarks of frontotemporal lobar degeneration (FTLD) postmortem. The TANK-binding kinase 1 gene (*TBK1*) is on the list of genes that can contribute to the development of FTD as well as the related neurodegenerative disease amyotrophic lateral sclerosis (ALS).

**Methods:**

In this study, using an array of clinical and neuropathological data combined with biochemical and proteomics assays, we analyze the *TBK1* splice-mutation (c.1340 + 1G > A) in a Swedish family with a history of FTD and ALS. We also explore the K63 ubiquitination landscape in post-mortem brain tissue and fibroblast cultures.

**Results:**

The intronic (c.1340 + 1G > A) mutation in *TBK1* results in haploinsufficiency and affects the activity of the protein in symptomatic and pre-symptomatic mutation carriers.

**Conclusion:**

Our results suggest that the mutation leads to a significant reduction of TBK1 activity and induce alterations in K63 ubiquitination profile of the cell already in the presymptomatic stages.

**Supplementary Information:**

The online version contains supplementary material available at 10.1007/s00415-021-10887-x.

## Background

Frontotemporal dementia (FTD) is a fatal neurodegenerative disease that primarily affects the frontal and temporal lobes of the brain, resulting in changes in personality, behaviour and/or language [1]. Onset of FTD is generally in the middle years of life with approximately 8 years of survival [2, 3]. From a neuropathological perspective, frontotemporal lobar degeneration (FTLD) is classified in three major categories of FTLD-TAU, FTLD-TDP and FTLD-FUS, based on detection of the respective protein in the intracellular inclusions. Nonetheless, a common feature among all groups is positive immunoreactivity of the inclusions to ubiquitin and p62 [4–6].

The current knowledge about the genetics of FTD suggests that the majority of genetically inherited FTD can be explained by a pathogenic mutation in *C9ORF72*, *MAPT* and *GRN* genes. Nevertheless, rare mutations in other genes, namely *TARDBP*, *VCP*, *CHMP2B*, *SQSTM1*, *UBQLN1* and *TBK1* are also reported to be causative for FTD [7]. To date, more than 70 variants in the *TBK1* gene have been reported as causative for FTD and/or ALS [8].

The TBK1 protein is proposed to function in multiple discrete pathways, among which regulation of autophagy and inflammatory signaling are the most well studied [9, 10]. Both signaling pathways remain relevant in the context of neurodegeneration, however, the autophagic pathway stands out, as it is a major protein quality control mechanism for the non-dividing neuronal cells [11, 12]. Mutations in genes encoding different components of the autophagic pathway namely Optineurin (*OPTN*) and Sequestosome-1 (*SQSTM1)* (coding for p62) are also associated with FTD and ALS. Moreover, many other genes that in one way or another are engaged with the components of the autophagy-lysosome machinery, (such as *GRN*, *VCP*, *C9ORF72*) can also explain the disease pathogenesis [13, 14].

Considering the regulatory role of TBK1 in autophagy and inflammatory signaling, it is fitting to review the molecular mechanisms of TBK1 activity. TBK1 is regulated by phosphorylation on S172 within the kinase activation loop. Serine-to-alanine substitution at this position abolishes TBK1 activity, whereas the phosphomimetic mutation S172E partially restores the protein’s function [15]. Analysis of the crystal structure of TBK1 suggests that recruitment of TBK1 to signaling complexes, occurs through interaction with distinct scaffolding proteins such as optineurin. This in turn results in TBK1-activation through proximity via trans autophosphorylation [16]. Some studies suggest that up to 70% of the TBK1 activity is the result of its interaction with the adaptor protein OPTN [17], while other studies have reported that TBK1 regulates the autophagy receptors OPTN and p62 during bacterial infection by phosphorylating the UBAN domain on these proteins [18–20].

Conjugation of ubiquitin moieties to the proteins result in different regulatory effects on the target protein. The ubiquitin itself can also be polymerized on lysine residues forming polyubiquitin chains. The K48 and K63 linkages are the most abundant form of polyubiquitination, accounting for approximately 80% of the total linkages in mammalian cells [21]. While K48-linked ubiquitin chains are mostly associated with proteasomal degradation, K63-linked ubiquitin chains regulate processes such as inflammatory signal transduction, DNA repair, endocytosis, mitophagy and selective autophagy [22, 23]. TBK1-mediated phosphorylation of OPTN results in increased affinity of the ubiquitin-binding domain of optineurin towards lysin-linked, K63-polyubiquitinated proteins, and therefore has strong implications for the role of TBK1 in regulation of mitophagy and determining the fate of the K63-ubiquitinated proteins [24]. Phosphorylation of p62 on Ser-403 also increases the affinity of p62 for K48- and K63-linked ubiquitin chains, and enhances the autophagic clearance of p62 and polyubiquitinated protein aggregates [20]. Previous studies have analyzed the effect of TBK1 loss of function in cells from a *TBK1*-null mouse model (Mouse Embryonic Fibroblasts, (MEF)), describing accumulation of LC3-II (a marker of early autophagic vesicles), suggesting that TBK1 facilitates the maturation of autophagosomes under basal conditions [19]. The same study also shows the accumulation of ubiquitinated proteins in *Tbk1* knockout MEFs using a TUBE (Tandem Ubiquitin Binding Entities) based purification. However, the nature and function of these proteins were never studied further.

The *TBK1* c.1340 + 1G > A (p.Ala417*) mutation was first identified in a Swedish family with a history of familial ALS [25]. The mutation leads to a substitution of Guanine to Adenine, in the first nucleotide of intron 11, resulting in skipping of exon 11[26]. Based on quantitative PCR analysis, and detection of approximately 50% expression of TBK1 RNA in mutation carrier cells, previous studies have generally concluded that the mutation results in a loss of function and have suggested that the mutant RNA is targeted for degradation by non-sense mediated RNA decay (NMD). This conclusion is further supported by Western Blot analysis using anti-TBK1 antibody, rendering haploinsufficiency as the proposed mechanism for the loss of function and pathogenicity of the *TBK1* p.Ala417* variant [25].

In this study, we describe the clinical phenotype, neuropathology and molecular effects of the *TBK1* p.Ala417* mutation in another, to our knowledge unrelated, Swedish family with multiple cases of FTD and ALS. The expression of *TBK1* p.Ala417* haploinsufficiency is explored in fibroblast cell-cultures and blood samples from asymptomatic mutation carriers as well as frontal cortex from an affected mutation carrier. Furthermore, we have explored the K63-ubiquitination landscape, in frozen postmortem brain tissue from a patient with *TBK1* p.Ala417*-FTD as well as in fibroblasts from pre-symptomatic mutation carrier and non-carrier donors. Interestingly we detect significant changes of the protein-ubiquitination profiles in tissue from both brain and fibroblast cultures with the *TBK1* mutation compared to control tissues. Our results suggest that effects of the *TBK1* haploinsufficiency, caused by the p.Ala417* mutation, start already in the pre-symptomatic stage and indeed affect important cellular regulatory mechanisms such as autophagy and K63 ubiquitination in mutation carriers, years before the onset of the disease.

## Materials and methods

### Family description

**Fig. 1:**
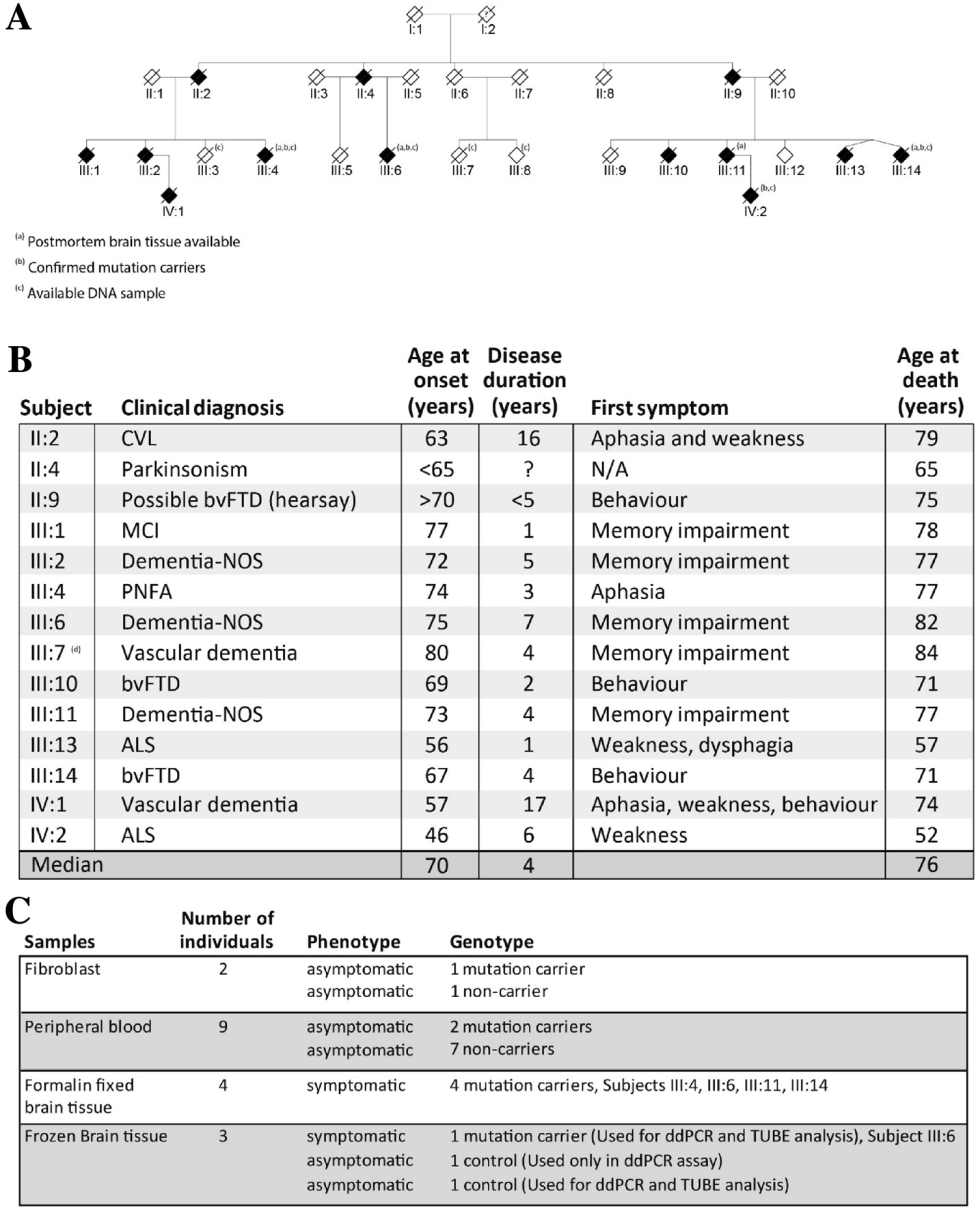
**A** Pseudonymized pedigree of the Swedish family with a history of hereditary FTD and ALS caused by the *TBK1* p.Ala417* mutation. **B** Table of demographic data for generations II–IV. **C** Representation of the number of individuals and the sample types used in the study as well as the genotype and phenotype of the donors. CVL, cerebrovascular lesion; bvFTD, behaviour variant frontotemporal dementia; MCI, mild cognitive impairment; NOS, not otherwise specified; PNFA, progressive non-fluent aphasia; ALS, amyotrophic lateral sclerosis; NA, not available. Marked by (**a**) indicate the individuals with available brain tissue. **b** Represent the individuals with confirmed mutation status. **c** Indicate the individuals with available DNA sample. **d** Indicate that subject III:7 is known to be a *TBK1* non-carrier

One large family with autosomal dominant FTD-ALS caused by a pathogenic mutation in *TBK1* was recruited via the Unit for hereditary dementia at Karolinska University Hospital in Stockholm, Sweden (Fig. 1). The family history of dementia, and related disorders, were collected through interviews, patient medical records and the Swedish National Archives. The clinical descriptions of patient phenotypes were obtained from medical records and reviewed by the authors (LÖ, CG) (Fig. 1B). Age at symptom onset was set to the age at which the first symptom was noticed by medical personnel or an informant. Peripheral blood, fibroblasts and brain samples were collected from a subset of the family members (Fig. 1C). Asymptomatic, at-risk individuals in the fourth generation (not shown in pedigree to keep anonymity) were invited to participate in the clinical and prospective GENetic Frontotemporal dementia Initiative (GENFI) study [27]. At risk individuals were assessed at the Unit for hereditary dementia (including medical and neuropsychological examinations and magnetic resonance imaging of the brain) and showed no signs of cognitive dysfunction all with a score equal to or above 26 on the Montreal cognitive assessment (MOCA). The mutation status of participants was assessed using PCR and Sanger sequencing as previously described by [26]. Genetic screening of two family members with two different phenotypes (one with ALS and one with bvFTD) was performed including several FTLD- / ALS-related genes (*C9ORF72*, *MAPT*, *GRN*, *VAPB*, *SOD1*, *TARDBP*, *SQSTM1*, *FUS*, *VPS131C*, *FLNC*, *TBK1*) and the only pathogenic variant detected was the *TBK1* variant p.Ala417*.The *TBK1* mutation was confirmed in affected subjects with available DNA (Fig. 1).

All the brain samples used in the study were provided by Karolinska Institutet brain bank. The two frozen brain samples that were provided as controls, were donated by male donors with the age of 67 and 76 years old and no history of dementia. The donors of the control brains exhibited no FTLD- or ALS-related histopathology at the time of death.

### Immunohistochemistry and neuropathology

After fixation in formaldehyde the brains were processed using a standard protocol for neurodegenerative diseases. Immunostainings were performed on superfrost slides of 5-µm-thick sections from the frontal and temporal lobes as well as from the hippocampus. For two patients, the medulla oblongata was also stained. For all patients, the commercially available antibodies to the following proteins were used: TDP-43 (1:5000, Proteintech); pTDP-43 (1:20,000, Cosmo Bio Co., Ltd), ubiquitin (1:50,000, Merck Millipore) and p62 (1:1000, Enzo Life Sciences). The immunostainings were performed in a Ventana BenchMark Ultra instrument.

### RNA, cDNA preparation and droplet digital PCR for gene expression

Peripheral blood was collected in PAXgene Blood RNA tubes and the RNA was extracted with RNAeasy Plus Micro Kit (QIAGEN, Hilden, De). All samples showed RIN > 7 on the Agilent 2100 Bioanalyzer. Furthermore, cDNA was generated using the QuantiTect Rev Transcription Kit (QIAGEN, Hilden, De).

Droplet digital PCR (ddPCR) was used to examine the *TBK1* cDNA levels. MIQE guidelines were followed for development and data analysis of digital PCR. The *TBK1* gene expression was determined by both relative (control gene RPL3) and absolute copies/µl using ddPCR. However, both approaches yielded similar results (data not shown) and absolute copies are presented. One [1] microliter of cDNA per sample was mixed with QX200 ddPCR Evagreen supermix (Bio-Rad, Hercules, CA) and primers amplifying both the *TBK1* exon 10_11 junction and *TBK1* exon 11. (*TBK1* Fwd atg att tag acg ggg atg ct and *TBK1* Rev ttc tga tac aga aat cca atg tga). Primers were designed based on the *TBK1* gene sequence: *TBK1* (ENST00000331710.10) and checked with the Transcript: NM_013254.3; Protein: NP_037386.1; UniProt: Q9UHD2 in Alamut^®^ Visual 2.11 (SOPHiA GENETICS, Lausanne, Swiss). Samples were assayed in triplicates in three independent experiments after optimization. PCR mixtures were emulsified in 1 nl droplets generating 20,000 droplets per sample in a QX100 Droplet generator (Bio-Rad) and using the droplet generator DG8 cartridge containing 20 μl of reaction mixture and 70 μl of QX200™ Droplet Generation Oil for EvaGreen per well (Bio Rad). Multiple negative reactions containing water mixed with the PCR reaction buffer were included on each plate. PCR amplification was performed in a Bio-Rad T100 Thermal Cycler as per manufacturer’s indications, with annealing temperature set at 56 °C and the ramp rate set at 2 °C. The fluorescence of the droplets was measured using QX200 droplet reader (Bio-Rad, Hercules, CA, US) and data was analyzed using the QuantaSoft™PRO Analysis Pro (Bio-Rad). Threshold was set manually against multiple negative controls consisting of ddPCR cocktail mixed with water as non-template control. The cluster corresponding to the mutant splice variant, resulting from the mutation c.1340 + 1G > A (p.Ala417*), was defined manually using the loop tool in the 2D plot generated with the QuantaSoft™PRO Analysis Pro. For this purpose, multiple mutant cDNA samples were compared against control samples with the reference allele.

### Skin biopsy and cell culture

Skin biopsies were taken from participants according to local protocols. Primary dermal fibroblasts were isolated, cultured and stored in – 130 °C cryo-freezers by the Clinical genetics facility at the Karolinska University hospital. Fibroblasts were thawed and cultured in Dulbecco's Modified Eagle Medium (Thermo Scientific) supplemented with 15% Fetal Bovine Serum, L-glutamine and non-essential amino acids.

### Cell lysis and immunoblotting

Fibroblasts were cultured to a confluency of 95% in T75 flasks. Cells were then washed two times with ice-cold PBS before harvesting for lysis. To prepare the protein lysate, RIPA lysis buffer (Thermo Scientific) complemented with Complete protease inhibitor cocktail (Roche), PhosSTOP™ (Roche) and 100 nM N-Ethylmaleimide (Merck) were used. Next, lysis buffer was added to the flask and cells were scraped off followed by 20-min incubation on ice. The lysate was then collected and sonicated three times for 20 s with 10 s breaks in between on ice. The lysates where then cleared by centrifugation at 20,000*g*. The cleared lysate was collected for further analysis. BCA protein Assay kit (Thermofisher) was used for protein concentration measurement. The protein samples were either used immediately or mixed with 4 × Laemmli Sample Buffer (Bio-rad), heated in 95 °C for 5 min, and stored in − 80 °C in aliquots. For TUBE pulldown experiments, as suggested by the TUBE bead manufacturers, 1,10-Phenanthroline and PR-619 (Lifesensors) were also added to the lysis buffer prior to the lysis.

Western blot analysis was performed according to standard protocols. Protein lysates were separated by Any kD SDS-PAGE gels (Bio-Rad) and transferred to a 0.2 μm PVDF membrane (Bio-Rad) using a semi-dry Trans-Blot Turbo transfer system (Bio-Rad). All membranes were blocked with 5% BSA in 1 × TBS (50 mM Tris − HCl, pH 7.5, 150 mM NaCl) for 1 h at RT and incubated with primary antibodies overnight at 4 °C. Primary antibodies were diluted in 1 × TBS with 3% BSA at the following dilutions: rabbit anti-TBK1 (Abcam) at 1:500, mouse anti-Tubulin at 1:4000 (Sigma-Aldrich), rabbit anti p-TBK1 (abcam) at 1:500 and rabbit anti-LC3B (Novus) at 1:500. The membranes were washed five times for 10 min in TBST (1 × TBS with 0.075% Tween-20), incubated with the appropriate NIR tagged secondary antibodies against the relevant host species at 1:10,000 (Licor) for 1 h and washed again five times 10 min, all at RT, before detection by Odyssey^®^ CLx imaging system (Licor).

### TUBE pulldown assay and mass spectrometry

The mass spectrometry (MS) was performed together with the Karolinska Institutet core facility for mass spectrometry-based proteomics (Solna, Sweden), similar to [28] and as described below.

The analysis was performed using three biological replicates of protein extracts from frontal cortex of an FTD brain sample (III:6 in Fig. 1A) and from a control brain sample alongside fibroblast protein extracts from a pre-symptomatic mutation carrier and from a non-carrier control. For each sample subjected to MS-based identification of K63 ubiquitinated proteins, Tandem ubiquitin binding entities (TUBE) in the form of Magnetic K63-TUBE beads (Lifesensors) were used as described according to the manufacturers protocol. K63 polyubiquitinated proteins from 2 mg of protein lysates from fibroblasts and post-mortem brain tissues were incubated with 100 µl of pre-equilibrated K63-TUBE magnetic beads in 20 mM Tris, pH 8.0, 0.15 M NaCl, 0.1% Tween-20 (TBS-T) with rotation overnight at 4˚C. Magnetic beads were separated using a magnetic stand and were washed three times with 1 × TBST. To perform the Mass spectrometry analysis, the washed beads were subjected to three extra steps of washing with TBS followed by on-bead digestion with Trypsin. The resulting peptides were injected onto an LC–MS/MS system (UltimateTM 3000 RSLCnano chromatography system and Q Exactive Plus Orbitrap mass spectrometer, Thermo Scientific), where they were separated on a homemade C18 column, 25 cm (Silica Tip 360 μm OD, 75 μm ID, New Objective, Woburn, MA, USA) with a 60-min gradient at a flow rate of 300 nl/minute. The gradient went from 5 to 26% of Buffer B (2% Acetonitrile, 0.1% Formic acid) in 120 min up to 95% of Buffer B in 5 min. The obtained peptides were identified by screening the MS data against the Uniprot KB database.

### Data analysis

The quality control and preliminary statistical analysis of the mass spectrometry data was done in Proteome Discoverer 2.4 (Thermo Scientific) (Supplementary Fig. 3A-B). The heatmaps and volcano plots were constructed using R (The R Foundation for Statistical Computing; version 4.0.2) and R Studio software (RStudio Team; version 1.2.5033) based on clustering analyses using agglomerative hierarchical clustering and Wards clustering criterion. The dissimilarity matrices were constructed using Pearson correlations.

In the case of missing values for specific features (proteins), a suitable substitute value was imputed via the following algorithm: first the number of missing values for that feature was checked across all replicates for each sample. If that number exceeded one (i.e., two out of three replicates do not have values), then that feature was excluded. Next, the algorithm checked if a peak was detected. If not, the feature was excluded. If a peak was detected, then the missing value was set to the minimum detected value divided by two, among the other two replicates for that feature that have a value. This was done separately for both the features identified in the K63 ubiquitination dataset obtained from the fibroblasts and the brain sample dataset.

Differential ubiquitination was assessed using a statistical t-test analysis, which identified those proteins with significantly altered abundance levels (*p* < 0.05) and a log2 fold difference of at least two between the control and mutation carrier replicates.

Gene Ontology terms for the biological processes associated with each identified protein were extracted and subjected to statistical clustering using the DAVID (Database for Annotation, Visualization and Integrated Discovery) functional annotation clustering tool.

## Results

### Clinical and neuropathological description of the TBK1 p.Ala417* FTD cases

Symptoms associated with FTD and/or ALS was observed in members of the family in three generations (Fig. 1A). The *TBK1* splice-mutation p.Ala417* segregates with disease and follows an autosomal dominant pattern with variable expressivity. In one branch of the family, the phenotypes are described as progressive non-fluent aphasia (PNFA, also called non-fluent variant of primary progressive aphasia, nfv-PPA), dementia-NOS (not otherwise specified), and parkinsonism while the phenotypes in the other branch includes both FTD as well as ALS, consistent with FTD-ALS. The mean age at onset of dementia was 70 years (range 57–80 years) whereas the two individuals with ALS had a younger age at onset, 46 and 56 years, respectively.

**Fig. 2:**
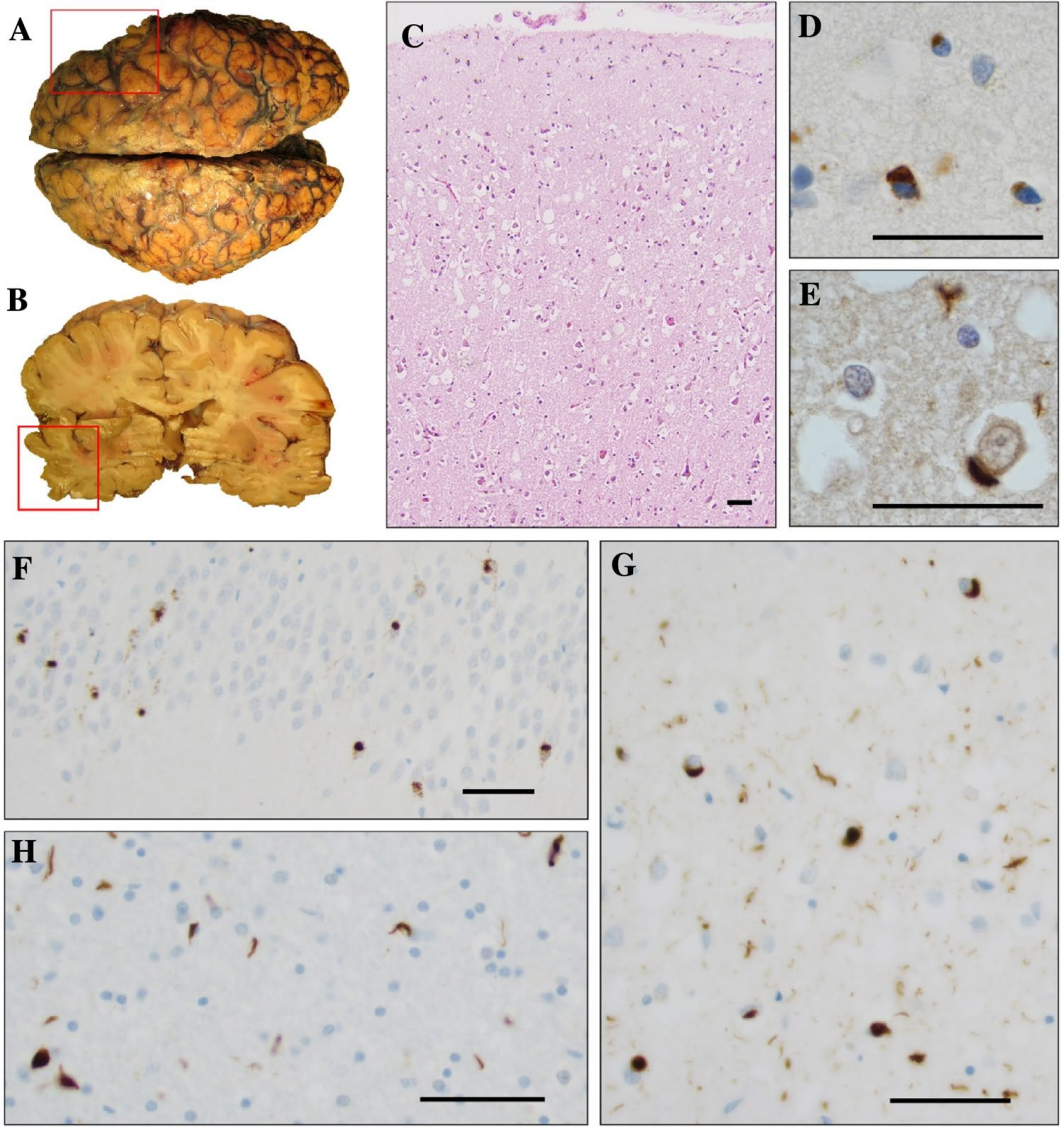
Representative images of the neuropathological examination of *TBK1* p.Ala417*-mutation carriers in the family in Figure A. **A**, **B** Superior view and coronal section of the brain. There is minimal amount of atrophy which is highlighted with the red box corresponding to the right temporal lobe. **C** Hematoxylin and eosin staining of frontal cortex with some superficial vacuolization. **D** p62 staining of frontal cortex. **E**) Ubiquitin staining of frontal cortex. **F** pTDP-43 staining of the granular cells of the dentate gyrus in hippocampus **G** pTDP-43 staining of frontal cortex. **H** pTDP-43 staining of the white matter. The scale bars represent 50 µm. Pictures are taken from three of the four postmortem cases: Panels **A** and **B** from one individual (diagnosed with Dementia-NOS), panels **C**, **F**, **G** and **H** are from a second individual (diagnosed with Dementia-NOS frontal) and panels **D**, and **E** are from a third individual with bvFTD

The clinical features of four individuals, where autopsy and postmortem neuropathology is also available, are presented here. A summary of the clinical diagnoses, ages at onset and ages at death of all affected individuals in the family is presented in Fig. 1B. A summary of the neuropathological examinations is presented in Fig. 2.

Subject III:4: onset of effortful speech with reduced fluency and word retrieval difficulties at the age of 74 years. Fulfils clinical criteria for PNFA [29]. Along with the language difficulties, motor symptoms (mainly spasticity and ataxia) were developed as the disease progressed and the patient died at the age of 77 years. Immunohistochemistry for TDP-43 and pTDP-43 showed a few intracytoplasmic inclusions in mainly superficially located neurons. Occasional neuronal lentiform intranuclear inclusions were also found. Positive short threads were present in the cortex and subcortical white matter. Some oligodendroglial cytoplasmic inclusions were seen. In the granular cell layer of the dentate gyrus, a few intracytoplasmic inclusions were found.

Subject III:6: onset of episodic memory dysfunction, impaired executive and visuospatial skills and mild behavioral symptoms at the age of 75 years. Due to slow progression, minor cortical atrophy and preserved functions of daily living, the symptoms were evaluated as mild cognitive impairment for many years. Subsequently, tremor and walking difficulties developed, which led to the suspicion of atypical parkinsonian disorder or motor neuron disease. Electromyography (EMG) showed affected peripheral neurons but not definitive MND/ALS. After 6 years of mild symptoms, the disease progressed, and the diagnosis was converted to Dementia-NOS. Death occurred at the age of 82 years and a neuropathological examination was performed. Immunohistochemistry for TDP-43 and pTDP-43 showed intracytoplasmic inclusions in mainly superficially located neurons. A few neurons had a lentiform intranuclear inclusion. Short positive threads were seen in the cortex and subcortical white matter. Some oligodendrocytes with intracytoplasmic inclusion were found. The pathology seemed to be more pronounced in the temporal lobe. Intracytoplasmic neuronal inclusions in the granular cell layer of the dentate gyrus were found. In the olivary nuclei of the medulla oblongata, positive threads and intracytoplasmic neuronal inclusions were present. Immunoreactivity was not observed in motor neurons of the hypoglossal nuclei.

Subject III:11: onset of memory problems and apraxia at the age of 73 years. Cognitive impairment in episodic memory, verbal fluency and executive function. Develops motor symptoms including rigidity, loss of facial expressions, slowness and tremor supporting a possible diagnosis of atypical parkinsonian disorder. Death occurred at age of 77 and a neuropathological examination was performed. Immunohistochemistry for TDP-43 and pTDP-43 showed frequent neurons with intracytoplasmic inclusions in mainly superficial cortical layers. Intranuclear lentiform inclusions were also present. Positive short threads were seen in the cortex and the subcortical white matter. Intracytoplasmic inclusions in many oligodendrocytes were also found. In the granular cell layer of the dentate gyrus, many neurons contained a positive intracytoplasmic inclusion. Occasional neuron had a lentiform intranuclear inclusion. In the inferior olives, nuclei in the medulla positivity was seen in threads and in neuronal intracytoplasmic inclusions. Immunoreactivity was not present in motor neurons of the hypoglossal nuclei.

Subject III:14: onset of an accelerating apathy and inertia at the age of 67 years. Also present with stereotypic behavior and disinhibition and therefore fulfils clinical criteria for behavioral variant FTD [30]. There was no clinical suspicion of motor neuron disease or parkinsonian disorder at any time in the disease course. The patient died at an age of 71 years. Immunohistochemistry for TDP-43 and pTDP-43 showed superficially located neurons with intracytoplasmic inclusions. Occasional neurons with intranuclear lentiform inclusion were found. Positive short threads were present in the cortex and subcortical white matter. Cytoplasmic oligodendroglial inclusions were found. In the granular cell layer of the dentate gyrus reactivity was present in the cytoplasm of some neurons.

For all cases, the TDP-43 immunohistochemical staining pattern corresponded to frontotemporal lobar degeneration-TDP type A pathology [31] and the staining pattern of pTDP-43 was similar to the ubiquitin and p62 staining (Fig. 02 D-H).

### TBK1^A417X^ carriers have decreased TBK1-expression and -activity

**Fig. 3:**
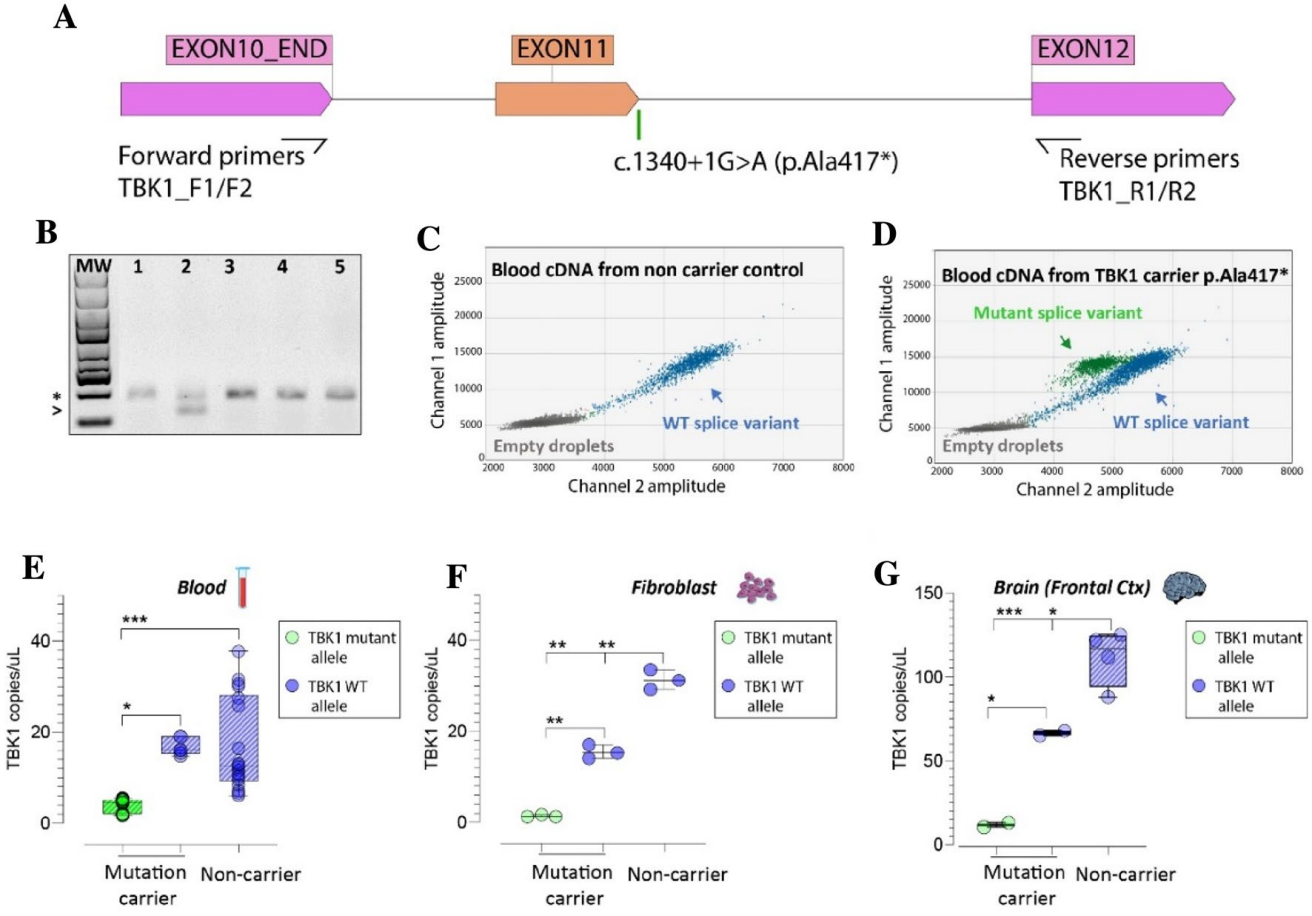
**A** Forward and reverse primers used in the *TBK1* transcript analyses illustrated in panels **B**–**G**. The amplicon contains the location of the *TBK1* c.1340 + 1G > A (p.Ala417*) mutation in intron 11. **B** Image of an agarose gel electrophoresis of cDNA extracted from blood in non-carriers (lanes 1, 3, 4, and 5) and a presymptomatic mutation carrier (lane 2). The asterisk indicates the wildtype transcript and the arrowhead indicates the shorter mutated transcript with an exon-11 deletion. **C**–**G** ddPCR of cDNA from blood (two presymptomatic mutation carriers and seven non-carriers), fibroblasts (one presymptomatic mutation carrier and one non-carrier) and frozen brain tissue (one mutation carrier with FTD and two non-carriers). Clusters are formed from the signals in single droplets: blue (wildtype transcript), green (mutated transcript) and grey (empty droplets). **C**, **D** Representative two-dimensional ddPCR plots. **E**–**G** Absolute number of transcript copies per microliter. Statistical differences were calculated by one-way ANOVA followed by Tukey’s Multiple comparison post hoc Test. Adjusted *p* values: *p < 0.020, **p < 0.0007, ***p < 0.005. Taken together our results confirm previously described haploinsufficiency of *TBK1* in affected mutation-carriers and we also show that the haploinsufficiency is present already at the presymptomatic stage

We have previously reported the p.Ala417*, (c.1340 + 1G > A) variant in the present Swedish family with multiple cases of FTD and ALS [26] (Fig. 3A). The mutation was first identified by [25] in another Swedish family with a history of familial ALS. The variant c.1340 + 1G > A is a point mutation in the splice-donor site in intron 11 which results in skipping of exon 11 during mRNA splicing and an out of frame stop-codon at amino acid position 417 (Fig. 3A).

For a more detailed analysis of the mutation in this family, we used samples from both presymptomatic mutation carriers (PMC) (blood and fibroblasts) as well as affected mutation carriers (AMC) (postmortem brain tissue) (Fig. 1C). As shown in Fig. 3B, the mutant *TBK1* blood-mRNA is incompletely degraded in the presymptomatic mutation carrier as determined by visual inspection of fragments and agarose gel electrophoresis. Freischmidt et al., 2015 showed similar results in patient-derived cells with this and other *TBK1* loss of function-mutations. They also reported a reduction of *TBK1* expression to ~ 50% as determined by qRT-PCR measurement of *TBK1* mRNA abundance in patient-derived cells [25]. We robustly confirmed this reduction in presymptomatic mutation carriers and assessed the absolute expression of the mutant and wildtype *TBK1* alleles. For this, ddPCR was used to quantify the levels of mutant and wildtype *TBK1*-transcripts in blood and fibroblasts obtained from PMC as well as brain, obtained from AMC and controls. As shown in Fig. 3 C-G, there is a significant reduction of the expression of the mutant allele compared to the wildtype allele in the PMC samples. The absolute expression follows as 4 ± 1.5 vs 16.7 ± 1.8 copies/µl (adjusted *p* < 0.02) in PMC blood, whereas, in PMC fibroblasts the values are 1.4 ± 0.2 vs 15.5 ± 1.5 copies/µl, (adjusted *p* < 0.0001) (Fig. 3 E–F). Similar pattern follows in AMC frontal cortex where there are 11.8 ± 2 vs 66.4 ± 2 copies/µl of the mutant vs the wildtype allele (adjusted *p* < 0.02) (Fig. 3G). Furthermore, we were not able to detect any sign of compensatory increased expression of the wildtype allele in response to the reduced mutant allele-expression, at least not in fibroblast and brain tissue (Fig. 3 F, G). Our data suggest a total decrease of *TBK1 RNA-*expression in mutation carriers which corresponds to 54% and 70% of the levels in controls, in fibroblasts and brain tissue respectively, indicating a chronic reduction in expression of *TBK1* starting in preclinical phases and extending to more severe FTD stages. (Fig. 3F, G).

**Fig. 4:**
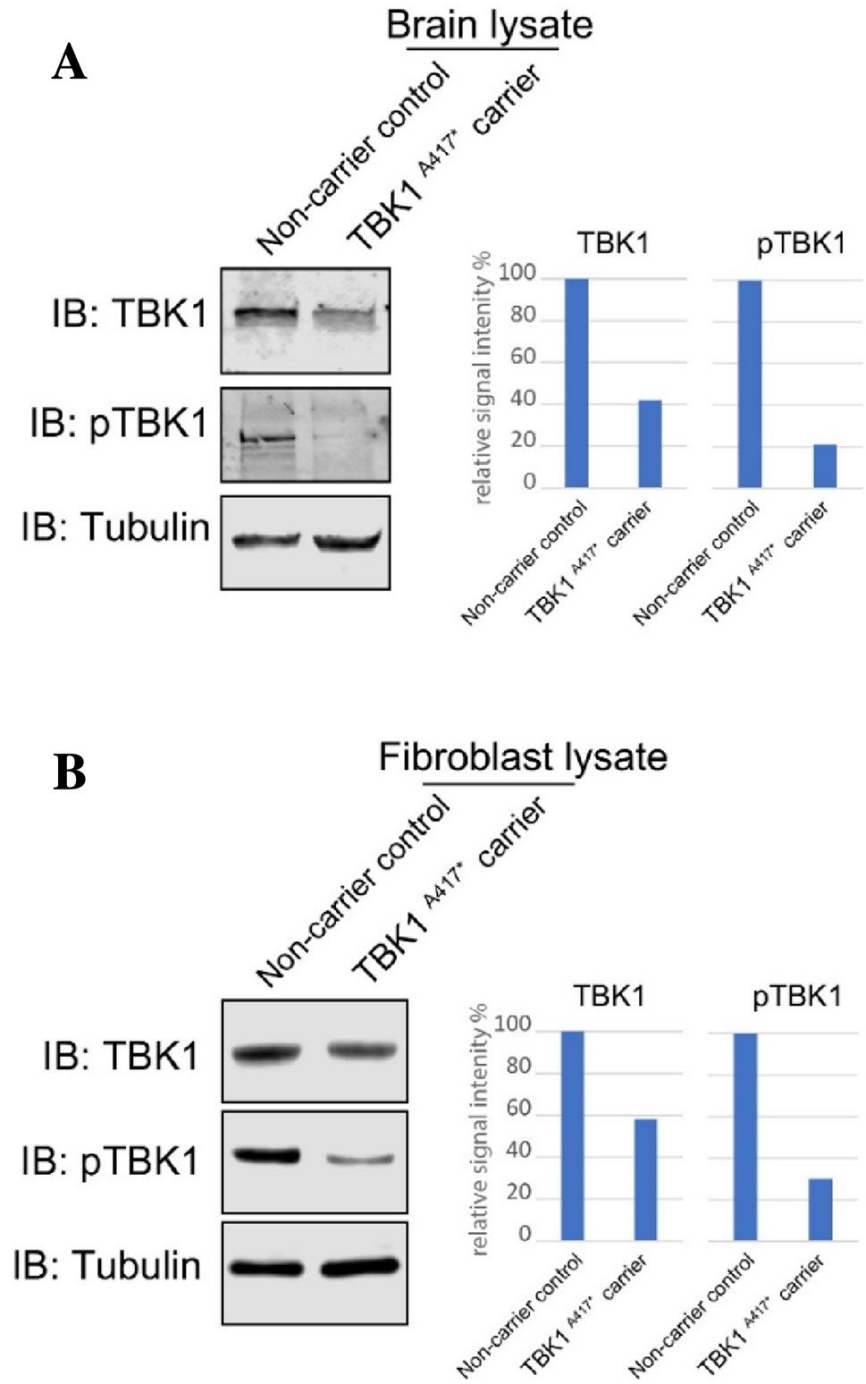
Western blot analysis of TBK1 expression; **A** Expression of TBK1 and pTBK1 is reduced in the brain lysates from an affected mutation carrier sample; **B** Expression of TBK1 and pTBK1 is reduced in the fibroblast sample from *TBK1* pre-symptomatic mutation carrier. Quantification of the band intensity relative to tubulin blot and normalized against the non-carrier sample is presented for each panel

We further show that both the levels of TBK1 protein and its active form, phospho-TBK1 (Ser172), are significantly reduced already at the presymptomatic stage in fibroblasts as well as in post mortem tissue of a mutation carrier (Fig. 4). This suggests that the reduced TBK1 expression and its activity (reflected by the levels of the phosphorylated form), is potentially a life-long stressor that occurs many years before the onset of symptoms in presymptomatic mutation carriers. Although we did not attempt to explore the effect of the *TBK1* mutation on autophagic flux, we did observe increased levels of LC3II in fibroblast-lysates from the *TBK1* p.Ala417* presymptomatic mutation carrier. This is indeed in agreement with the role for TBK1 in the maturation of autophagic organelles, however, we were not able to detect a similar effect in a brain sample from an affected mutation carrier (Supplementary Fig. 2), suggesting a certain degree of tissue specificity in this regard.

### TBK1 haploinsufficiency alters the K63 ubiquitination profile of the cell

Previously, it was established that *Tbk1* knockout results in accumulation of polyubiquitinated proteins in MEFs [19]. To study the latter in our samples, we designed a TUBE-based proteomics assay and analyzed the K63 ubiquitination landscape of brain and fibroblasts. Using label free mass spectrometry analysis of the TUBE purified K63 ubiquitinated proteins we identified 2234 proteins in brain and 952 proteins in fibroblasts (Supplementary Tables 1 and 2, respectively). From the total number of identified proteins 371 (13.1%) occurred in both sample types, and the remaining proteins were consequently found solely in brain or fibroblast samples. (Supplementary Fig. 03C).

**Fig. 5:**
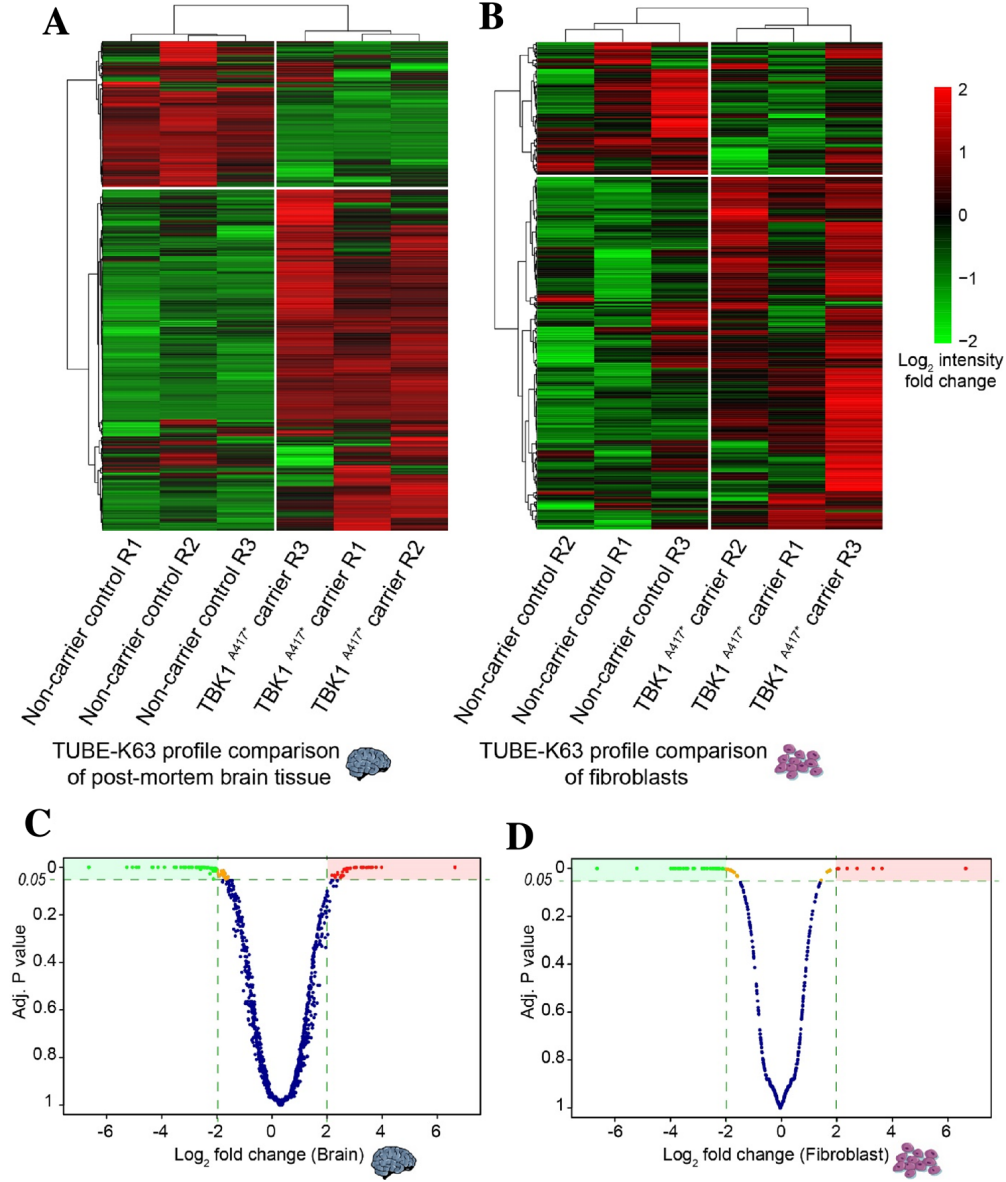
Analysis of the K63 ubiquitination landscape of *TBK1* mutation carriers and controls in fibroblasts and brain. **A**, **B** Heatmap representing the log2 fold-change values of differentially K63-ubiquitinated proteins in *TBK1* mutation carrier samples vs. control samples. Proteins and samples are separately clustered via hierarchical clustering. Proteins are colored based on the intensity of detected signal post TUBE-purification with green representing a fourfold decrease and red representing a fourfold increase in the detected signal relative to the mean signal of the protein across all replicates of each genotype. **C**, **D** Volcano plots of proteins in brain (**C**) and fibroblasts (**D**) identified by mass spectrometry according to their Benjamini–Hochberg adjusted **p** value (*y* axis) and their relative abundance ratio (log2 fold change) (*x* axis). Red dots represent the proteins detected with increased levels in mutation carrier samples and green dots represent the proteins detected in higher levels in control samples. Orange dots represent proteins that do not fulfill the cutoff of log2 fold difference. Dotted lines present the p value cutoff of *p* < 0.05 and log2 fold difference

As shown in dendrograms in Fig. 5 A and B, these identified proteins were able to correctly cluster the samples by mutation status. Furthermore, as depicted in Heatmaps, there are two clusters of proteins in all mutation carrier- and control-replicates in which we detect a relative increased or decreased K63-ubiquitination. These clusters consist of 615 proteins (23.1%) from the brain dataset and 208 proteins (21.8%) from the fibroblast dataset that display decreased K63 ubiquitination, and 1440 proteins (64.5%) from the brain dataset and 550 proteins (57.8%) from the fibroblast dataset that exhibit a relative increased K63 ubiquitination (Fig. 5 A-B). Comparability of the different replicates were ascertained by plotting the overall detected signal for all features. (Supplementary Fig. 3 A, B).

In brain samples, of the total number of identified proteins, 168 proteins were significantly differentially ubiquitinated, whereas, in fibroblasts, 109 proteins were differentially ubiquitinated. The latter is illustrated in Volcano plots in Fig. 5, describing the ratio of the protein abundances between the two different genotypes and the Benjamini–Hochberg adjusted p value for each identified protein (Fig. 5C, D). Interestingly, five of these hits, overlap between the brain tissue and fibroblast samples. (Supplementary Fig. 3D).

To map the major functional categories to which the differentially ubiquitinated proteins belong, we employed a compilation of the gene ontology terms associated with the identified proteins. Using the functional annotation clustering tool (DAVID), the identified differentially ubiquitinated proteins were grouped into several functional clusters. The analysis revealed 21 GO terms grouped in 6 major functional clusters for detected proteins in the fibroblast dataset and 19 GO terms grouped into 6 major functional clusters for the detected proteins in the brain dataset. A summary of the gene ontology analysis against the whole proteome is presented in supplementary Fig. 4. The major clusters that are highlighted in both fibroblast and brain samples are the GO terms associated with protein expression regulatory mechanisms as well as cell–cell adhesion proteins. However, when we limit the background GO dataset to cover only the identified K63 ubiquitinated proteins, the only significant hit corresponds to the cluster with mitochondrial respiratory chain (*p* < 0.05).

## Discussion

In this report, we put the knowledge of *TBK1* haploinsufficiency from the initial analysis into a new perspective by studying the specific *TBK1* c.1340 + 1G > A (p.Ala417*) mutation in depth in both clinical and molecular aspects. Interestingly we observe a certain degree of consistency in the results from post-mortem brain tissue and samples from pre-symptomatic mutation carriers.

The clinical presentation of neurodegenerative disease in this family varies with regards to age at onset, initial symptom and phenotype. From the third generation, the family is divided into two main branches. Patients in one of the branches (right side of pedigree in Fig. 1A) present predominantly with FTD or ALS while the phenotypes in the other branch is more heterogeneous. The varying presentations might be due to complicating factors in diagnosing dementia such as comorbidity (e.g., cardio-vascular-related symptoms). We could also speculate that the diversity potentially is a result of variable penetrance of the *TBK1* mutation and genetic-modifiers but this is not yet known.

At the cellular level, it has been proposed that TBK1 activation and substrate specificity are driven by recruitment to discrete signaling complexes or adaptor proteins such as p62 and OPTN that regulate its subcellular localization [10, 16, 17]. High local concentration of TBK1 or phosphorylation by other kinases that are localized to the same molecular scaffold are the critical parameters when it comes to regulation of TBK1 activity. Therefore, a reduction in the expression of *TBK1* potentially result in changes in the stoichiometric dynamics of TBK1 recruitment by adaptor proteins and thereby affect overall activation of the kinase and cause a chronic chain reaction of downstream stressors. TBK1 is also needed to enable the entry of p62 into autophagic degradative pathway, and several ubiquitinated cargos do not enter degradative pathways in the absence of TBK1 [20]. Interestingly, despite their diverse clinical phenotypes, the neuropathological examinations of four of the family members showed similar pattern with all having TDP-43 type A pathology and positive aggregates of p62 and ubiquitin which overlap with the staining’s from pTDP-43 aggregates.

K63 ubiquitination has been associated with formation and clearance of protein inclusions in neurodegenerative diseases [32]. At the cellular level, dysregulation of the cellular protein homeostasis- and protein degradation-machineries such as selective autophagy and the Ubiquitin Proteasome System (UPS) are among the main candidate causative mechanisms of neurodegeneration in FTD [33]. We were able to identify specific patterns regarding increase and decrease of K63 ubiquitinated proteins in response to mutation in *TBK1*. These results suggest that even though TBK1 is not a direct regulator of the ubiquitination machinery, it can indirectly influence the overall K63-ubiquitination profile of the cell in dermal fibroblasts and brain tissue. More importantly the changes to the ubiquitination pattern seem to occur many years before the onset of the disease and are already detectable in peripheral cells such as fibroblasts in presymptomatic mutation carriers.

Taken together, these findings imply that using TUBE based profiling of ubiquitination can be a valuable tool to study the neurodegenerative diseases. Similar attempts on ubiquitination profiling have already been done for Alzheimer disease (Tramutola et al., 2018) [34]. We believe that this analysis is an initiative to put more focus on the importance of the ubiquitination profiling as a potential marker for deciphering the underlying cause of the neurodegenerative diseases such as FTD or ALS.

An important question that our study raises is about the fate and nature of these alternatively ubiquitinated proteins. It remains unclear whether these proteins contribute to the overload to the cellular protein quality control systems or if they act as a primer to form protein aggregates that result in neuronal cytotoxicity through aging. We attempted to tackle this question using a gene ontology profiling of our list of differentially K63-ubiquitinated proteins for each examined tissue. Even though we believe drawing robust conclusions from gene ontology analysis is not feasible, due to the diverse nature of gene ontology terms and small sample size, we are able to see specific patterns emerging in functional clustering of GO terms that fit the theme of protein expression and regulatory mechanisms. To further validate our gene ontology analysis, we made a separate GO-analysis and instead if using the genome as reference we restricted the background/reference to the pool of K63-ubiquitinated proteins that we were able to detect and looked for gene ontology terms that are occurring only in the significantly altered ubiquitinated proteins. Interestingly the cluster referring to mitochondrial respiratory chain stand out as statistically significant (*p* < 0.05), which is in line with previous reports about the role of *TBK1* in regulation of mitophagy [35].

Given the small number of available samples and the lack of material from individuals who have developed the disease, we were unable to expand the ubiquitination study in the affected individuals beyond the post-mortem brain tissue. Based on what we have observed in terms of diversity between ubiquitination landscape of fibroblast and brain tissue is not possible to draw decisive conclusions. Nonetheless, the significance of the ubiquitination differences in response to *TBK1* loss of function remains relevant. Overall, we hope that this study lays a foundation for future studies in the underlying consequences of *TBK1* haploinsufficiency and the mechanisms of its pathogenicity.

## Supplementary Information

Below is the link to the electronic supplementary material.

Supplementary file1 (XLSX 374 KB)

Supplementary file2 (XLSX 158 KB)

Supplementary file3 (DOCX 855 KB)

## Data Availability

The authors confirm that the data supporting the findings of this study are available within the article [and/or] its supplementary materials.

## Abbreviations

FTD: Frontotemporal dementia
NA: Not available
UPS: Ubiquitin proteasome system
ALS: Amyloid lateral sclerosis
FTLD: Frontotemporal lobar degeneration
TBK1: Tank binding kinsase 1
UBAN: Ubiquitin binding in ABIN and NEMO
PCR: Polymerase chain reaction
ddPCR: Digital droplet PCR
MEF: Mouse embryonic fibroblast
TUBE: Tandem ubiquitin binding entity
NMD: Non-sense mediated decay
GENFI: GENetic frontotemporal dementia initiative
MS: Mass spectrometry
TBS: Tris-buffered saline
TBST: Tris-buffered saline, tween
RNA: Ribonucleic acid
DNA: Deoxyribonucleic acid
cDNA: Complementary DNA
mRNA: Messenger RNA
NIR: Near-infrared
PNFA: Progressive non-fluent aphasia
nfv-PPA: Non-fluent variant primary progressive aphasia
NOS: Not otherwise specified
bvFTD: Behavioural variant FTD
MND: Motor neuron disease
PMC: Presymptomatic mutation carrier
AMC: Affected mutation carrier
DAVID: Database for annotation, visualization and integrated discovery
TDP-43: TAR DNA-binding protein 43
CVL: Cerebrovascular lesion
GO: Gene ontology
