# Supplementary material for: TBK1 haploinsufficiency results in changes in the K63-ubiquitination profiles in brain and fibroblasts from affected and presymptomatic mutation carriers
Source: J Neurol. 2021 Nov 20;269(6):3037–49. doi: 10.1007/s00415-021-10887-x (PMC9120096; doi:10.1007/s00415-021-10887-x)
Supplement: Supplementary file 3 — Supplementary file3 (DOCX 855 KB) [file 415_2021_10887_MOESM3_ESM.docx]

***Supplementary figures***


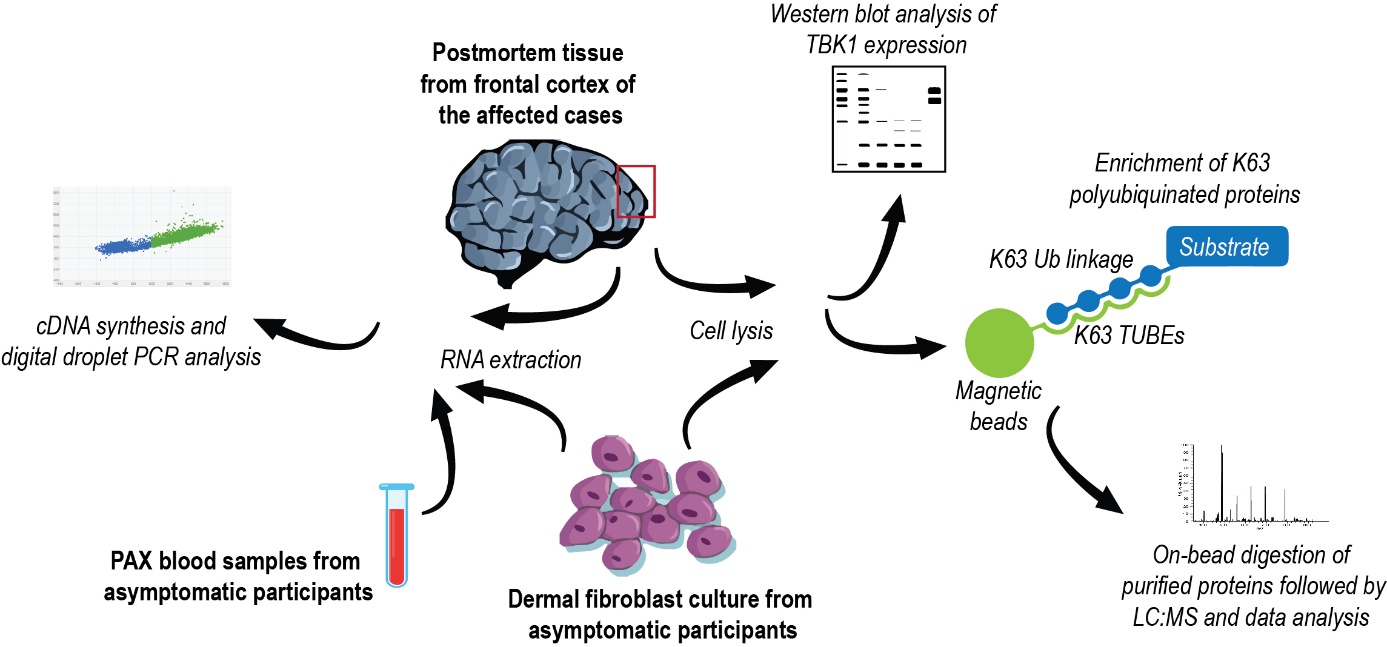


Figure S01, An overview of the methodology of the study.


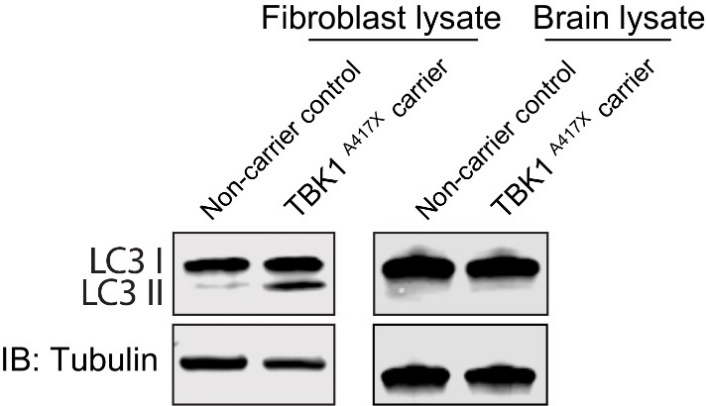


*Figure S02, Analysis of LC3II levels in brain and fibroblast of TBK1 mutation carrier samples. Western blot analysis shows that increased levels of LC3II is detectable in fibroblasts from pre-symptomatic mutation carrier (fibroblasts) compared to the control, however, a similar effect is not detected in the brain sample from an affected mutation carrier.*


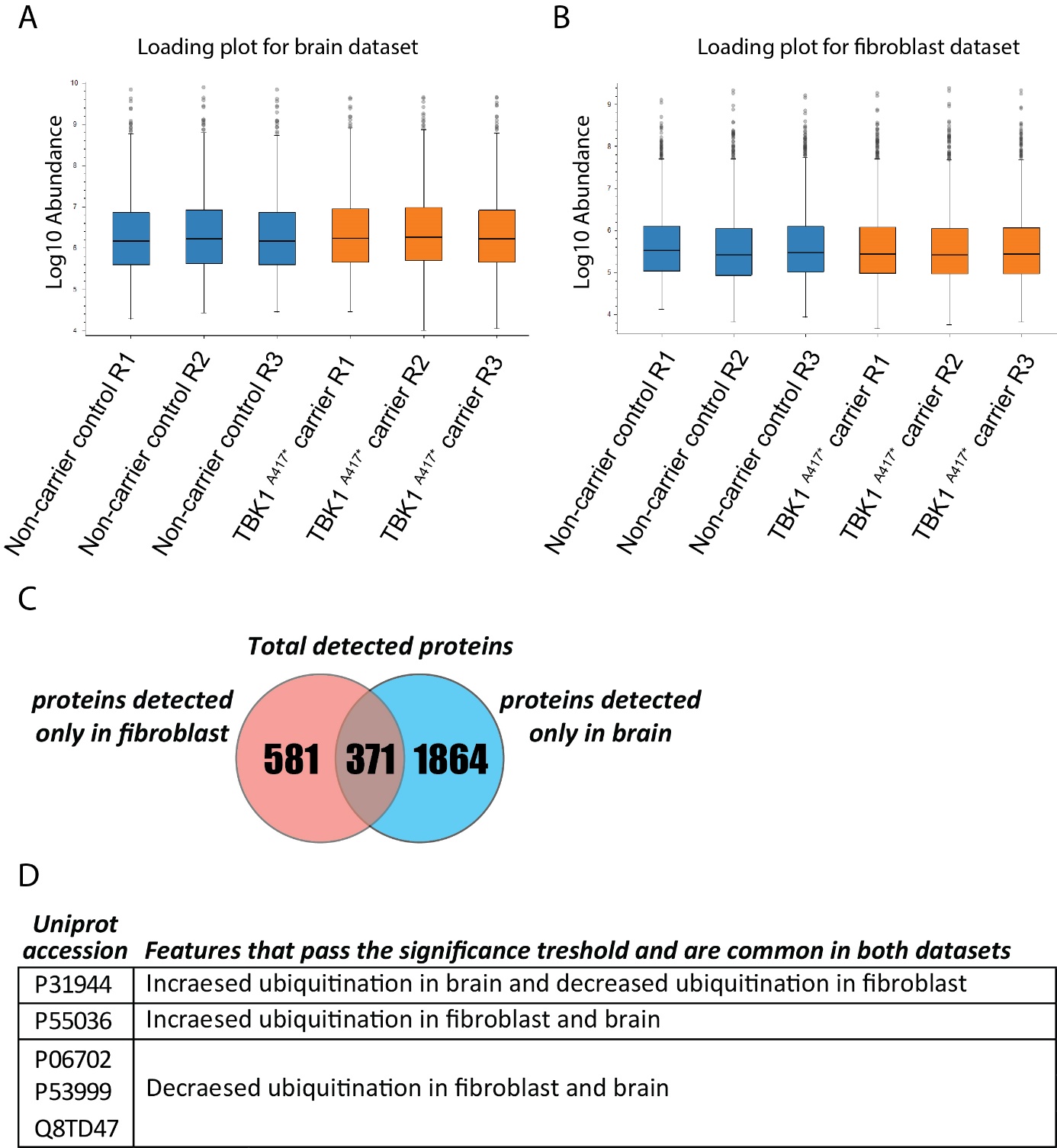


Figure S03, A) Loading control for the mass spectrometry data. Box plots represent the median of Log10 abundance of the total detected proteins. 50 percent of the peptide intensities are between the 25th and 75th percentile lines and whiskers represent the 5% and 95% percentile of the signal intensities. B) The Venn diagram of the overlap between the identified proteins in fibroblast and brain datasets. C) The overlap of the proteins that display a significant increase or decrease of K63-ubiquitination in brain and fibroblast databases. D) Table of features that are within the significance threshold and are found in both datasets.


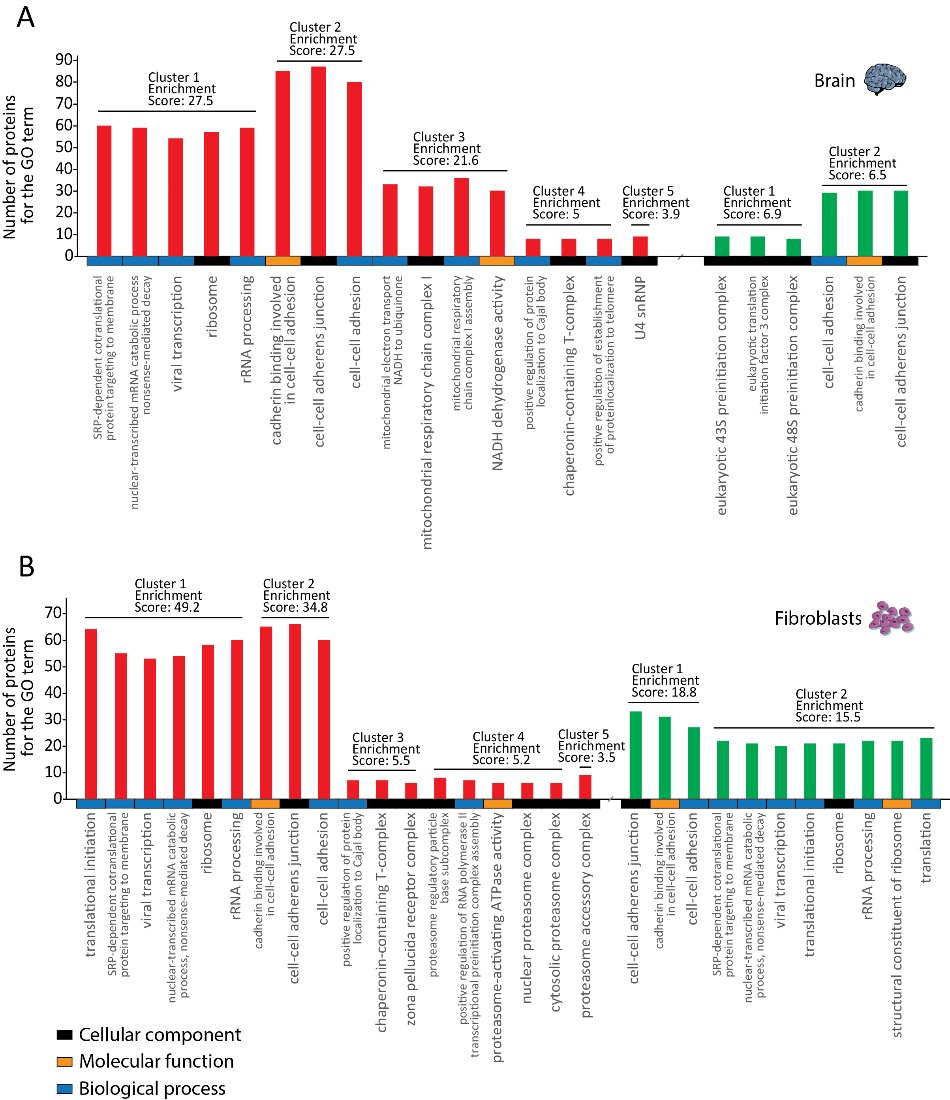


Figure S04, Functional characterization of the identified K63 ubiquitinated proteins using the DAVID database, suggesting that the differentially K63 ubiquitinated proteins are involved in multiple different biological processes. Enrichment scores of >3.5 were considered as meaningful.
